# IgM–C4d Complex Causes the Inaccurate Measurement of Serum Uric Acid Levels via the Uricase Method

**DOI:** 10.3390/cimb48070657

**Published:** 2026-06-25

**Authors:** Yuexinzi Jin, Yuan Mu, Li Wang, Bingfeng Zhang, Suli Ge, Huaguo Xu, Jian Xu, Jiexin Zhang

**Affiliations:** 1 Department of Laboratory Medicine, The First Affiliated Hospital with Nanjing Medical University, Nanjing 210029, China; jinyuexinzi@njmu.edu.cn (Y.J.);; 2 Branch of National Clinical Research Center for Laboratory Medicine, Nanjing 210029, China

**Keywords:** uric acid, uricase method, immunoglobulin M, complement component 4d, mass spectrometry

## Abstract

Accurate measurement of serum uric acid (UA) is critical for disease assessment and therapeutic monitoring; however, numerous factors can compromise the accuracy of UA detection. This study describes a novel circulating immunoglobulin M (IgM)-involved protein complex that interferes with the uricase method and reduces serum UA measurement accuracy. A total of 24 serum samples were collected from 18 patients, and complete clinical information and laboratory data were obtained. Samples were divided into three groups according to their UA reaction curves. Optical density values were extracted to analyze differential insoluble properties, serum viscosity was measured, coimmunoprecipitation was performed for IgM complex detection, and three additional clinical methodologies were used for UA measurement and comparison. All samples exhibiting unique reaction curves showed simultaneous elevations in IgM and complement component 4 (C4) levels. Circulating IgM formed a protein complex with C4d without significantly increasing serum viscosity. The insolubility of the IgM–C4d complex was attributable to the particular alkaline component of the uricase reagent. Inaccurate UA measurements could only be corrected by mass spectrometry. This study represents the first report of the circulating IgM–C4d protein complex. Given that serum UA serves as a crucial therapeutic indicator for specific patient populations, mass spectrometry is the preferred analytical method for accurate UA quantification in these individuals.

## 1. Introduction

Uric acid (UA) is a waste product of purine metabolism. Approximately 2/3 of UA is eliminated via urine, and the other half is eliminated via feces [1]. When the serum level of UA exceeds the amount that the kidney and intestine can metabolize, it is deposited in soft tissues in the form of urate crystals that can cause gouty arthritis [2]. Studies have indicated that an elevated serum UA level is closely related to the occurrence and development of chronic kidney diseases, autoimmune diseases, and cardiovascular diseases [3,4,5].

At present, serum UA can be measured via UA sensors, spectrophotometry, high-performance liquid chromatography, mass spectrometry, the phosphotungstic acid reduction method, and the uricase method [6]. The American Association of Clinical Chemistry recommends the liquid reagent-based uricase method as a secondary reference method. Owing to its high specificity and sensitivity, as well as its simpler procedure that does require deproteinization, the uricase method is ideal for use in automatic biochemical analyzers in clinical laboratories [7]. However, several factors interfere with the accuracy of the uricase method. Unsaturated bonds of vitamin C reduce the intermediate product of hydrogen peroxide, which results in a decrease in the measured UA concentration [8,9]. Other endogenous substances, such as glutathione, catalytically active transitional metal ions, and xanthine, have analogous effects [10,11,12]. Rasburicase is a recombinant urate-oxidase enzyme approved by the US Food and Drug Administration to control blood UA levels in both pediatric and adult patients, especially in those with tumor lysis syndrome (TLS) [13]. It effectively catalyzes the oxidation of UA to allantoin both in vivo and ex vivo.

Owing to the monoclonal malignant proliferation of plasma cells or B lymphocytes, masses of immunoglobulin (Ig) accumulate in the blood to form “paraproteins” in patients diagnosed with multiple myeloma and macroglobulinemia, and high serum viscosity is observed in these patients [14,15]. Multiple factors, including pH, buffer components, and ionic strength of the reagent, are critical for decreasing the solubility of paraproteins, which can increase serum turbidity and decrease detection accuracy [16]. In this study, we identified a new IgM-involved protein complex that interferes with the uricase method for the measurement of serum UA levels. Alternative methods for correcting serum UA measurements in clinical practice were compared and discussed.

## 2. Materials and Methods

### 2.1. Patient Population

We screened patients in our hospital between 1 October 2023, and 31 December 2024, and the inclusion criteria were (1) high serum IgM concentration and (2) at least one simultaneous measurement of serum IgM, IgG, IgA, complement component 3 (C3), C4, and UA levels. The exclusion criteria were as follows: (1) treatment with drugs to reduce UA levels (e.g., vitamin C, glutathione, rasburicase, and mercaptopurine) before blood draw; and (2) incomplete measurement data for serum immunological markers and UA. A total of 24 samples from 18 patients (12 males and 6 females, ages ranging from 35–77 years old) between 1 October 2023, and 31 December 2024, were collected. This study was approved by the Ethics Committee of the First Affiliated Hospital of Nanjing Medical University (Nanjing, China).

### 2.2. Measurement of Serum UA Levels with an Automatic Biochemical Analyzer via the Uricase Method

The serum UA levels were measured via the uricase method in an automatic biochemical analyzer (AU5800, Beckman Coulter, Brea, CA, USA). System calibrators (Cat. No. 66300, Beckman Coulter) were used to adjust the instrument’s response to ensure accurate and reliable measurement results of the analytes, and the assayed chemistry control (C-310-5, Bio-Rad, Hercules, CA, USA) was applied to monitor the stability and reliability of the instrument’s testing process and results during routine operation.

The detection kit contains two liquid reagents, namely Reagent 1 and Reagent 2. Reagent 1 comprises the following components: 6.8 g/L K_2_HPO_4_, 0.175 g/L 3,5-dimethylaniline disodium salt (MADB), and 5 KU/L ascorbate oxidase. Reagent 2 comprises the following components: 1.84 KU/L uricase, 45.6 KU/L peroxidase (POD), and 0.46 g/L 4-aminoantipyrine (4-AAP). For each sample, Reagent 1, the sample, and Reagent 2 are added sequentially to the same reaction cup. UA is oxidized into allantoin and hydrogen peroxide via uricase. Hydrogen peroxide together with and MADB and 4-AAP is then catalyzed by POD to generate chromophores. The chromophores are measured at wavelengths of 660 nm and 800 nm. A total of 27 measurements is performed (from “point 0” to “point 27”), with each measurement separated by 18 s, yielding 27 optical density (OD) values. In specific, after 36 μL of Reagent 1 was added to a reaction tube and placed in an AU5800 instrument, the OD value was obtained and referred to as “point 0”. Next, 4.2 μL of each serum sample was added, mixed and incubated with Reagent 1, and the OD values were continuously obtained from “point 1” to “point 9”. Reagent 2 was added to the mixture at “point 10”, and the OD values were continuously obtained until “point 27”.

To calculate serum UA concentration, a two-step formula was adopted. First, the volume correction factor *K* was determined (Equation (1)).

(1) $$K=\frac{V_{R1}+V_{S}}{V_{R1}+V_{R2}+V_{S}}$$

Subsequently, the OD value for UA concentration calculation was derived using *K* (Equation (2)).

(2) $$OD={OD}_{27}-K\times {OD}_{10}$$

where:

*K*: Intermediate correction factor for reagent and sample volume ratio;

*V*_R1_: Reagent 1 volume (μL);

*V*_R2_: Reagent 2 volume (μL);

*V*_S_: sample volume (μL);

OD: Optimal density for UA calculation;

OD_27_: Optimal density measured at point 27;

OD_10_: Optimal density measured at point 10.

To determine the effect of the alkaline component (K_2_HPO_4_) in Reagent 1 on the change in OD values at “point 0” and “point 1”, Reagent 1 was replaced with deionized water or a 6.8 g/L K_2_HPO_4_ solution. The serum samples were then analyzed in an AU5800 instrument to obtain OD values at “point 0” and “point 1”.

### 2.3. Measurement of Serum Viscosity

The viscosity of serum samples was measured using a ZL9600C Automated Hemorheology Analyzer (Zhongchi Ltd., Beijing, China), a dual-system viscometer integrating cone-plate technology for non-Newtonian fluids (e.g., whole blood) and capillary pressure-sensing technology for Newtonian fluids (e.g., serum/plasma). After 0.8 mL of each serum sample was added to a tube in an instrument based on the capillary flow method under a constant temperature of 37 °C, the viscosity was detected, and the output value was documented.

### 2.4. Measurement of Serum Immunoglobulins and Complements

We collected the data for serum immunoglobulins, including IgA, IgG and IgM, and complement components C3 and C4, which were measured via the rate nephelometric method in an automatic instrument (IMMAGE 800, Beckman Coulter, Brea, CA, USA) instrument.

### 2.5. Ex Vivo Coimmunoprecipitation

Three additional serum samples from three patients, drawn on the same day, were used for the coimmunoprecipitation assay: one with high IgM and C4 concentration (labeled No. 1), one with high IgA and C4 concentration (labeled No. 2), and one with high IgG and C4 concentration (labeled No. 3). The three patients had relatively similar serum C4 concentrations. Serum samples were kept on ice throughout all procedures. A 50 μL aliquot of Protein A/G magnetic beads (B23201, Selleck Chemicals, Houston, TX, USA) was transferred to a 1.5 mL microcentrifuge tube and washed three times with binding buffer (50 mM Tris, 150 mM NaCl, 0.1–0.5% Triton X-100, pH 7.5). A rabbit anti-human IgM antibody (ab212201, Abcam, Cambridge, UK) or control rabbit IgG (A7016, Beyotime, Shanghai, China) was diluted in binding buffer to a concentration of 50 μg/mL; then, 200 μL of the antibody solution was incubated with the beads with rotation at room temperature for 15 min to facilitate coupling.

For immunoprecipitation, both neat serum and serum diluted 50-fold in saline were separately incubated with anti-IgM antibody-coated beads for 1 h at room temperature. This step was designed to selectively pull down IgM molecules, along with any serum proteins bound to IgM. The immunocomplexes were washed three times with wash buffer, and the bound proteins were eluted using 30 μL of SDS loading buffer under reducing conditions. The eluted mixture was then heated at 100 °C for 5 min to disrupt protein–protein interactions and denature proteins, after which the samples were subjected to electrophoresis on a 10% SDS polyacrylamide gel.

For Western blotting, mouse anti-human C4 (sc-271181, Santa Cruz Biotechnology, Dallas, TX, USA, molecular weight: 98 kDa) and rabbit anti-human IgM (predicted band size: 49 kDa and observed band size: 78 kDa) were used as primary antibodies. HRP-conjugated goat anti-mouse IgG (A0216, Beyotime, Shanghai, China) and goat anti-rabbit IgG (A0208, Beyotime, Shanghai, China) served as secondary antibodies. Protein bands were visualized using a chemiluminescence detection system.

### 2.6. Correction Test

To determine the degree of inaccuracy of the uricase method in measuring serum UA levels, the polyethylene glycol (PEG) precipitation method, the dry slide-based method, and mass spectrometry were used, and the results were compared.

For the PEG precipitation method, 100 μL of 25% *w*/*v* PEG 6000 (Cat. No. 81255, Sigma, St. Louis, MO, USA) was added to 100 μL of each serum sample [17]. After intense vortexing, the mixture was centrifuged at 13,000× *g* for 15 min at room temperature. The supernatant was measured with an AU5800 instrument. The OD values were obtained at both 660 nm and 800 nm to calculate the UA concentration.

For the dry slide-based method, 10 μL of each serum sample was added to a slide with a VITROS 5600 (Ortho, Rochester, NY, USA) instrument. After incubation at 37 °C for 5 min, the OD value was obtained at a wavelength of 670 nm to calculate the UA concentration.

For mass spectrometric quantification of UA in serum, a calibration curve was prepared using a UA standard (CAS 69-93-2, Y11946, Beijing Biolab Science and Technology, Beijing, China). This standard was serially diluted in acetonitrile to generate a concentration range starting from 10 μg/mL. Serum samples were maintained on ice throughout processing. After vortexing at 1000 rpm for 5 min and centrifugation at 13,000× *g* for 15 min, 100 μL of supernatant was collected from each sample. The supernatant was diluted with 50 μL of purified water, followed by the addition of 450 μL of extraction solvent. Liquid–liquid extraction was performed by incubating the mixture at 4 °C for 1 h, then centrifuging at 12,000× *g* for 10 min. The resulting supernatant was evaporated to dryness under a stream of nitrogen, reconstituted in 1000 μL of appropriate solvent, and centrifuged again at 12,000× *g* for 5 min. Finally, the extract was diluted 10-fold and analyzed using a Qtrap 6500+ LC–MS/MS system (AB Sciex, Framingham, MA, USA). Quantification of UA was based on the external calibration curve.

### 2.7. Statistical Analysis

The data were analyzed using GraphPad Prism 8.0 (GraphPad, San Diego, CA, USA; https://www.graphpad.com/scientific-software/prism/ (accessed on 8 September 2025). The data are presented as $\bar{x}$ ± SD. Group differences were examined using ANOVA. The significance level was set at *p* < 0.05.

## 3. Results

### 3.1. Serum UA Measurement Inaccuracies in Some Patients with High Serum IgM Concentration

The serum UA levels in 14 samples collected from 9 patients (6 males and 3 females, ages ranging from 40–77 years old), between 1 October 2023, and 31 December 2024, were measured inaccurately. The main diagnosis was Waldenström macroglobulinemia, which occurred in 5 patients. Other diseases included diffuse large B-cell lymphoma in 1 patient, mature small B-cell lymphoma in 1 patient, extranodal marginal zone B-cell lymphoma in 1 patient, and lacunar infarction in 1 patient. The serological characteristics of the 14 samples were as follows: 4 (28.58%) had elevated IgM levels, 5 (35.71%) had simultaneous increases in IgM and complement component C 4 levels, and 5 (35.71%) had simultaneous increases in IgM, C4, and C3 levels.

**Figure 1:**
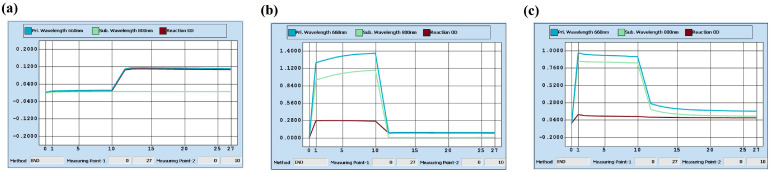
Reaction curves produced in the uricase method for serum UA measurement. (**a**) Accurate serum UA measurements. The calculated UA concentration was 417 µmol/L. (**b**) Inaccurate serum UA measurements in one serum sample from a patient with Waldenström macroglobulinemia with an elevated IgM level (IgM 36.3 g/L; reference range 0.3–2.2 g/L in males). The calculated UA concentration was −567 µmol/L. (**c**) Inaccurate serum UA measurements in one serum samples from a patient with Waldenström macroglobulinemia with simultaneous increases in IgM and C4 levels (IgM 17.9 g/L, C4 0.522 g/L; reference ranges: IgM, 0.3–2.2 g/L in males; C4, 0.1–0.4 g/L). The calculated UA concentration was 1 µmol/L.

Figure 1a shows a typical reaction curve produced in an accurate serum UA measurement. Figure 1b,c show reaction curves produced in inaccurate serum UA measurements using samples from two patients with Waldenström macroglobulinemia. The reason for the inaccurate results was that the OD values at “point 1” were much higher than those at “point 27”.

### 3.2. Different Interfering Effects of High Serum IgM Concentration on Serum UA Measurement Accuracy

**Figure 2:**
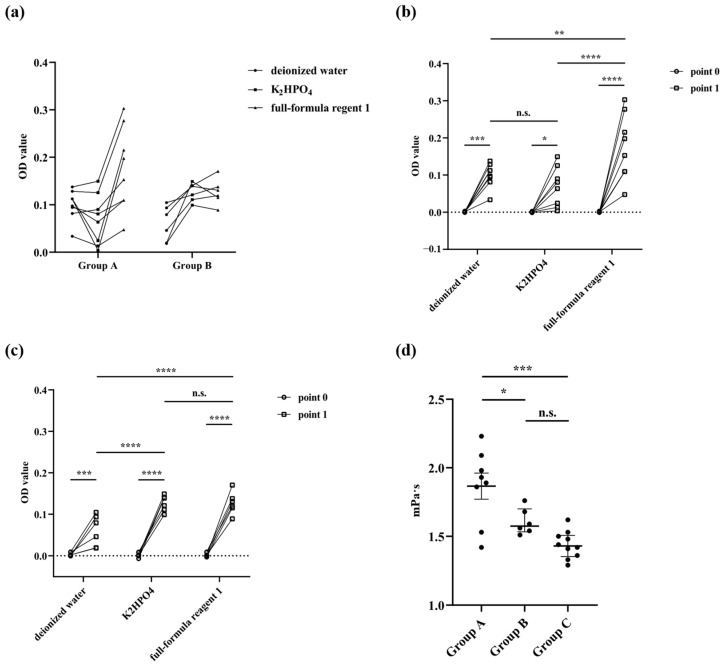
Differential characteristics of hyper-IgM serum. (**a**) Comparative changes in paired OD values at “point 1” in groups A and B. (**b**,**c**) Changes in the OD values at “point 0” and “point 1” in group A (**b**) and group B (**c**). (**d**) Serum viscosity values of the three groups. * *p* < 0.05; ** *p* < 0.01; *** *p* < 0.001; **** *p* < 0.0001; n.s., not significant.

We successively mixed each of the 14 serum samples with deionized water, 6.8 g/L K_2_HPO_4_ solution, or the full-formula regent 1 and compared the OD values at “point 0” and “point 1”. Based on the overall characteristics of OD value changes observed when serum samples were mixed with each of the three solutions, the 14 samples were divided into two groups (Figure 2a). There were 8 serum samples in group A and 6 serum samples in group B (Table 1).

Group A included eight serum samples from six patients. Compared with those at “point 0”, the OD values at “point 1” significantly increased when the serum samples were mixed with full-formula reagent 1 (0.0010 ± 0.0004 vs. 0.1765 ± 0.0312, *p* < 0.0001) (Figure 2b). The changes in the OD value after mixing with deionized water (0.0010 ± 0.0004 vs. 0.0996 ± 0.0114, *p* = 0.0002) were similar to those after mixing with a 6.8 g/L K_2_HPO_4_ solution (0.0006 ± 0.0004 vs. 0.0688 ± 0.0187, *p* = 0.0104) (Figure 2a, left panel and Figure 2b).

There were 6 serum samples in group B, which were from patients with IgM concentrations ranging from 13.4–18.4 g/L and high C4 levels (>0.4 g/L). The OD values at “point 1” obtained by mixing the serum samples with the 6.8 g/L K_2_HPO_4_ solution (0.1265 ± 0.0079) were greater than those obtained by mixing with deionized water (0.0602 ± 0.0153, *p* < 0.0001) and were similar to those obtained by mixing with full-formula reagent 1 (0.1271 ± 0.0110, *p* < 0.0001) (Figure 2a, right panel and Figure 2c). Notably, patient 7 had 3 serum samples (Table 1). The first serum sample was obtained from patient 7 when his IgM level was 17.9 g/L and his C4 level was 0.522 g/L, and the sample was placed into group B. Two months later, the serum IgM and C4 levels of patient 7 reached 29.6 g/L and 1.10 g/L, respectively. The sample taken at this time point was also in group B according to the characteristics of the reaction curve.

We also set a control group, group C, in which the IgM concentrations were between 2.33 g/L and 8.81 g/L, the C4 concentrations were all greater than 0.4 g/L, and the UA reaction curves were normal (Table 1). The serum viscosity values of the three groups were compared, and the results are shown in Figure 2d. The serum viscosity values were highest in group A (1.87 ± 0.10 mPa·s), and there was no significant difference between the other two groups (group B: 1.61 ± 0.04 mPa·s; group C: 1.44 ± 0.03 mPa·s).

### 3.3. Discovery of the Serum IgM–C4d Complex

**Figure 3:**
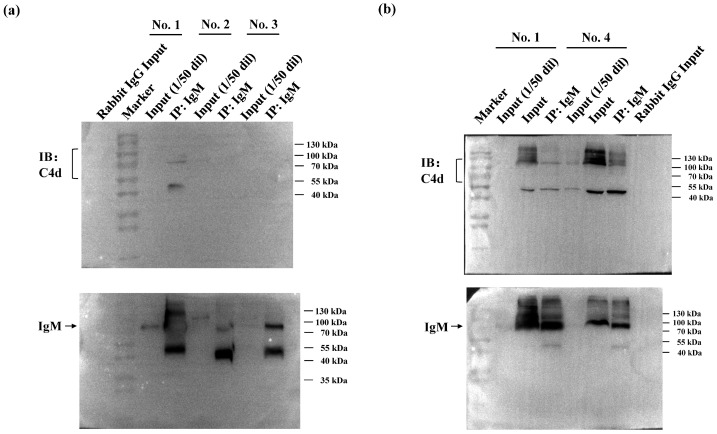
Identification of the IgM–C4d complex via coimmunoprecipitation. (**a**) No. 1: IgM, 17.5 g/L; IgA, 1.22 g/L; IgG, 10.5 g/L; C4, 0.543 g/L; C3, 1.18 g/L; No. 2: IgM, 0.586 g/L; IgA, 4.24 g/L; IgG, 13.9 g/L; C4, 0.480 g/L; C3, 1.15 g/L; No. 3: IgM, 1.10 g/L; IgA, 2.95 g/L; IgG, 18.2 g/L; C4, 0.413 g/L; C3, 1.00 g/L. (**b**) No. 1: IgM, 17.5 g/L; IgA, 1.22 g/L; IgG, 10.5 g/L; C4, 0.543 g/L; C3, 1.18 g/L; No. 4: IgM, 2.33 g/L; IgA, 1.23 g/L; IgG, 11.4 g/L; C4, 0.500 g/L; C3, 1.26 g/L. Reference ranges: IgM, 0.3–2.2 g/L for males, 0.5–2.8 g/L for females; IgA, 1.0–4.2 g/L; IgG, 8.6–17.4 g/L; C4, 0.1–0.4 g/L; C3, 0.7–1.4 g/L. Serum samples labeled as No. 1, No. 2, and No. 3 are detailed in “Materials and Methods”. Serum sample labeled as No. 4 corresponds to sample 17 in Table 1. “Input” represents that neat serum or serum samples diluted 50-fold in saline were added with SDS loading buffer in a 1:4 ratio (buffer: sample volume) for Western blot analysis.

A serum C4 detection kit (Roche Diagnostics, Indianapolis, IN, USA) was used in our clinical laboratory to detect full-length C4. Unexpectedly, IgM coimmunoprecipitated with C4d when the serum IgM and C4 levels were both elevated (Figure 3a). C4d is a stable degradation fragment derived from complement C4 after cleavage of its α-chain by activated C1s, followed by further proteolysis. More interestingly, the amount of the IgM–C4d complex decreased as the concentration of IgM increased (Figure 3b).

### 3.4. Methodological Comparison of Serum UA Measurements

Two commonly used methods of correction, the dry slide-based method and the PEG precipitation method, were chosen to measure serum UA levels. Mass spectrometry was used as the reference method. As shown in Table 2, both the dry slide-based method and the PEG precipitation method were able to correct the reaction curves and the UA concentrations. However, according to the newly released EFLM biological variation (BV) database, the higher limit of the interindividual BV of UA is 8.5 [18], which is significantly lower than the variation between the results of each correction method and those of mass spectrometry (Table 2).

## 4. Discussion

IgM has the greatest molecular weight among all immunoglobulins and diverse biological functions, including sterilization, complement activation, immune regulation, and agglutination in human blood [19,20]. Serum IgM levels in patients with Waldenström macroglobulinemia are closely related to the degree of tumor cell differentiation [21]. A significantly high IgM level (>45 g/L) is an independent predictor of symptom burst in smoldering Waldenström macroglobulinemia [14]. IgM-high myeloma is an infrequent subtype of myeloma that is characterized by high serum viscosity, lymphadenopathy, and hepatosplenomegaly [22].

Both a membrane-bound form of IgM (mIgM) and a secreted form of IgM (sIgM) are produced. MIgM consists of two homologous dimeric heavy chains, with each connecting to a light chain by a disulfide bond [23]. The μ-region of the heavy chain folds into four domains to allow the anchoring of mIgM to the surface of B cells, followed by the activation of C1 [24]. SIgM circulates in the form of a pentamer, which contains five IgM monomers and an additional small polypeptide J chain [25], enabling ten potential antigen binding sites [26] as well as interfaces with C1q and C3b [27].

In this study, we discovered that circulating IgM specifically formed a protein complex with C4d in a manner that was strictly dependent on the IgM dosage (Table 1 and Figure 3). C4 plays an indispensable role in the activation of classical and lectin complement cascades, both of which activate C2 to promote the formation of the membrane attack complex [28]. In the classical pathway, activated C1s cleaves complement C4, and subsequent degradation of C4b generates the stable fragment C4d [29]. Elevated serum IgM and C4 levels have been simultaneously observed in various pathological conditions [30]. Primary biliary cholangitis (PBC), an autoimmune disease characterized by anti-mitochondrial antibodies, is associated with elevated serum IgM due to IgM hyperresponsiveness and increased serum C4 resulting from excessive hepatic production in response to inflammation [31,32]. Proliferative glomerulonephritis (PGN) refers to a broad group of native renal diseases, such as lupus nephritis (LN) and membranoproliferative glomerulonephritis (MPGN). In one study conducted by Raman et al., immunohistochemistry was performed on renal biopsy samples from 107 PGN patients, and the samples from the LN (48 patients) and MPGN (14 patients) patients exhibited strong expression of both IgM and C4d [33]. Similarly, glomerular C4d deposition frequently co-localizes with IgM in other native kidney diseases, including IgA nephropathy, Henoch–Schönlein purpura nephropathy, and focal segmental glomerulosclerosis [34,35]. In addition, C4d deposits are consistently found alongside IgM isotype donor-specific antibodies in acute humoral renal and cardiac allograft rejection [36,37,38,39].

This is the first report of the presence of a serum sIgM–C4d complex. An increase in serum viscosity is not obvious, and the feature of this protein complex is entirely different from that of the classical “paraproteins”. The alkaline solution has a profound influence on the solubility of the sIgM–C4d complex, resulting in the inaccurate measurement of serum UA (Figure 2). It has been reported that factors influencing protein stability include temperature, salt type and concentration, solution pH, and binding ligands [40]. First, all patients’ serum samples appeared clear, indicating that any insolubility observed occurred during measurement with an automatic biochemical analyzer via the uricase method. Second, the same kit was used to measure serum UA from Group A and Group B, thereby eliminating potential interference from factors such as temperature, salt type, and salt concentration. Third, the OD values of the two groups exhibited different variation characteristics during UA measurement. When deionized water was added, the OD value range of Group A was wider than that of Group B (Figure 2b,c), suggesting that the solubility of IgM-formed complexes differs depending on the type of complex. In contrast, when an alkaline solution was added, the OD values of Group B changed more drastically compared to those after deionized water addition (Figure 2c), indicating that the IgM-C4d complex was more sensitive to changes in solution pH. This phenomenon is presumably associated with nonspecific repulsions between charged groups on proteins, which can destabilize the proteins’ folded conformations and trigger the insolubility of the IgM-C4d complex [40].

Abnormally increased serum IgM levels may decrease the accuracy of the uricase method. There is no unified method for the removal of such paraproteins, and satisfactory corrective effects have not yet been achieved [41,42]. However, it is recommended to pretreat samples via dilution, PEG precipitation, and ultrafiltration [43,44,45]. A dry film-based method enables the generation of a serum diffusion layer with characteristic capillary network structures for paraprotein segregation followed by the generation of a reaction layer. In this study, we compared the serum UA concentrations obtained via both the PEG precipitation method and the dry film-based method. Although the results obtained using the two methods were similar, their methodological biases to mass spectrometry were far beyond acceptance (Table 2). Mass spectrometry is formally recommended as the gold standard technique for measurement of blood 25-hydroxyvitamin D, an organic small molecule compound, in clinical laboratories [46]. Immunological methods, which are widely used for blood 25-hydroxyvitamin D measurement in clinical laboratories, are frequently interfered with various blood-borne metabolites (e.g., C3-epimer). These metabolites either exhibit structural similarity to 25-hydroxyvitamin D or share partial epitopes with it; as a result, reagents in the detection kit can cross-react with these metabolites, resulting in a bias up to −47% to mass spectrometry [47].

Accurate measurement of serum UA levels is essential for disease assessment and treatment. Chimeric antigen receptor-T (CAR-T)-cell therapy is now widely used for the treatment of hematologic malignancies, including refractory and relapsed multiple myeloma and B-cell lymphoma [48,49]. TLS is a frequently observed complication during CAR-T-cell therapy, and TLS development is associated with a significant decrease in overall survival [50]. In a cohort of 105 patients receiving CAR-T-cell therapy, Zhang et al. reported that 18 (17.1%) developed TLS with a median time to onset of 8 days (range 4–14), all of whom developed renal dysfunction [51]. Compared with patients without TLS, those with TLS had significantly higher baseline serum UA levels (428 μmol/L vs. 313 μmol/L, *p* < 0.01) and baseline creatinine (92.5 μmol/L vs. 68.0 μmol/L, *p* < 0.01) and significant shorter median progression-free survival and median overall survival.

Shely and Ratliff reported a 55-year-old patient with hematologic malignancy who received his first two doses of carfilzomib therapy and developed TLS [52]. On the fourth day of receiving carfilzomib, his serum UA level increased to 15.9 mg/dL (945 μmol/L). Rasburicase was promptly administered to decrease the patient’s serum UA to 0.3 mg/dL (17.8 μmol/L). This case demonstrated a complete picture of hyperuricemia. Falsely low serum UA levels delayed the diagnosis of TLS-related hyperuricemia, which resulted from the rapid destruction of tumor cells and massive release of intracellular components, leading to acute renal insufficiency, arrhythmia, epilepsy, and sudden death [53].

Therefore, in patients with elevated baseline UA or impaired renal function, serum UA should be closely monitored before, during, and after treatment. Laboratory technicians should be alert to erroneous reaction curves due to sIgM–C4d complex interference, and clinicians should be aware that concurrent elevation of serum IgM and C4 may cause falsely decreased UA values. In such cases, mass spectrometry can be considered as an alternative to obtain true UA levels and guide urate-lowering therapy.

In China, mass spectrometry has been successfully applied to the detection of nutrient indicators, endocrine-related indicators, inherited metabolic disease-related indicators, and polypeptide indicators, supported by well-established reference materials and external quality assessment programs [54,55]. However, there is currently no blood UA detection kit compatible with mass spectrometry. When utilizing mass spectrometry for hematological indicator detection, we propose a tiered testing strategy to address the cumbersome sample pretreatment procedures and limited maximum sample throughput per batch: Tier 1, screen for samples with high serum IgM and C4 levels; and Tier 2, perform serum UA measurement using mass spectrometry, thereby ensuring accurate results for specific patient populations.

We first described a new circulating IgM–C4d complex. A relatively high level of the IgM–C4d complex (13.4 g/L ≤ IgM ≤ 18.4 g/L and C4 > 0.435 g/L in this study) interferes with serum UA measurement accuracy through a distinctive mechanism, characterized by the concurrent presence of both water and alkali insolubility. We suggest mass spectrometry as the preferred method for patients with high levels of serum IgM and C4.

## Figures and Tables

**Table 1:** Information of the patients recruited for this study.

Group Name	Patient ID	Gender	Age	Sample ID	IgM (g/L)	C4 (g/L)	C3 (g/L)	UA-Original (µmol/L)	UA-MS (µmol/L)
Group A	Patient 1	male	76	sample 1	20.2	0.404	1.09	−47	315
				sample 2	21.4	0.194	0.989	−46	302
	Patient 2	male	76	sample 3	22.7	0.916	1.51	194	778
				sample 4	21.6	0.951	1.48	−105	427
	Patient 3	male	53	sample 5	36.3	0.155	1.04	−567	377
	Patient 4	male	65	sample 6	17.9	0.290	1.33	−164	465
	Patient 5	female	54	sample 7	37.4	0.347	1.39	11	283
	Patient 6	female	40	sample 8	35.5	0.415	1.69	−738	343
Group B	Patient 7	male	77	sample 9	17.9	0.522	1.18	1	291
				sample 10	29.6	1.10	1.90	133	362
				sample 11	17.4	0.666	1.38	19	360
	Patient 8	female	68	sample 12	13.4	0.435	0.986	−14	417
				sample 13	15.7	0.455	0.960	−13	442
	Patient 9	male	77	sample 14	18.4	1.16	2.05	15	431
Group C	Patient 10	male	53	sample 15	2.96	0.941	1.06	180	297
				sample 16	2.66	0.857	0.940	235	338
	Patient 11	male	76	sample 17	2.33	0.500	1.26	347	435
	Patient 12	male	35	sample 18	2.58	0.417	1.14	337	446
	Patient 13	female	49	sample 19	4.41	0.423	1.04	211	301
	Patient 14	male	75	sample 20	2.33	0.442	0.918	321	387
	Patient 15	female	53	sample 21	3.02	0.446	1.48	233	336
	Patient 16	male	74	sample 22	8.81	0.593	1.02	261	443
	Patient 17	female	52	sample 23	3.07	0.447	1.67	603	779
	Patient 18	male	76	sample 24	2.43	0.425	1.43	264	470

Reference ranges: IgM, 0.3–2.2 g/L for males, 0.5–2.8 g/L for females; C4, 0.1–0.4 g/L; C3, 0.7–1.4 g/L.

**Table 2:** The biases obtained from measuring serum UA concentrations via three methods.

Sample ID	Concentration by Mass Spectrometry (µmol/L)	Dry Slide		25% (*w*/*v*) PEG 6000	
		Corrected Concentration (µmol/L)	Methodological Bias to Mass Spectrometry (%)	Corrected Concentration (µmol/L)	Methodological Bias to Mass Spectrometry (%)
Sample 9	291	261	−10.3	250	−14.0
Sample 10	362	300	−17.1	280	−22.6
Sample 11	360	272	−24.4	258	−28.3
Sample 12	417	350	−16.0	336	−19.4
Sample 13	442	361	−18.3	340	−23.0
Sample 14	431	354	−17.8	332	−22.9

## Data Availability

The original contributions presented in this study are included in the article. Further inquiries can be directed to the corresponding authors.

## Abbreviations

The following abbreviations are used in this manuscript:

BV	Biological variation
C	Complement
CAR-T	Chimeric antigen receptor-T
Ig	Immunoglobulin
OD	Optical density
PEG	polyethylene glycol
TLS	Tumor lysis syndrome
UA	Uric acid
